# Safer conception for female sex workers living with HIV in Dar es Salaam, Tanzania: Cross-sectional analysis of needs and opportunities in integrated family planning/HIV services

**DOI:** 10.1371/journal.pone.0235739

**Published:** 2020-07-21

**Authors:** Eileen A. Yam, Catherine Kahabuka, Gaspar Mbita, Koheleth Winani, Louis Apicella, Caterina Casalini, Zuhura Mbuguni

**Affiliations:** 1 Population Council, Washington, DC, United States of America; 2 CSK Research Solutions Ltd., Dar es Salaam, Tanzania; 3 Sauti Program, Jhpiego, Dar es Salaam, Tanzania; 4 Reproductive and Child Health Section, Ministry of Health, Community Development, Gender, Elderly and Children, Dar es Salaam, Tanzania; 5 Population Council, Dar es Salaam, Tanzania; Children's Mercy Hospitals and Clinics Department of Pathology and Laboratory Medicine, UNITED STATES

## Abstract

**Background:**

With the advent of effective treatment, women living with HIV can plan for pregnancy while minimizing risk of transmission to infants and seronegative partners. Family planning (FP) services tend to focus solely on addressing contraceptive need, but HIV-positive women—including female sex workers—often plan to have children someday. Various “safer conception” strategies are now available to support women living with HIV achieve fertility intentions, and integrated HIV/FP services may be a promising platform to offer these services.

**Methods:**

At integrated community-based HIV/FP service delivery sites operated by Jhpiego’s Sauti project in Dar es Salaam, we conducted exit interviews with 300 HIV-positive female sex workers. Descriptive analyses were conducted to describe their desire for children, use of condoms and other modern contraceptive methods, self-reported viral suppression, and knowledge of and interest in safer conception strategies. We conducted bivariate and multivariate logistic regression analysis to examine correlates of fertility desire among respondents.

**Results:**

Median age of participants was 32. Nearly one-third wished to have a child within two years. Seventy-two percent had heard of having the HIV-positive partner taking ART to reduce sexual transmission during pregnancy attempts. Thirty-one percent felt the amount of FP content covered in the consultation was “too little.” Factors significantly associated with desire for children were having a nonpaying partner (adjusted odds ratio [AOR] 2.18, 95% confidence interval [CI]1.13–4.20) and having fewer children (AOR 0.65, 95% CI 0.48–0.87). Viral suppression was not associated with fertility desire.

**Conclusions:**

Sex workers living with HIV attending integrated HIV/FP services have need for both contraception as well as safer conception counseling. This integrated service delivery modality is a promising platform for providing safer conception services. FP counseling for HIV-positive women should be broadened to broach the topic of safer pregnancy, as well as explicit counseling on strategies to minimize risk of sexual transmission to partners.

## Introduction

### Background/Rationale

The advent of effective antiretroviral treatment (ART) has ushered in an era in which women living with HIV can lead full and healthy lives, including having children if they so desire [1–3]. Studies around the world consistently demonstrate that many women living with HIV—between 20% and 50%—want to have children [3–7]. There now is an array of biomedical and behavioral “safer conception” strategies to help these women get pregnant, while minimizing risk of onward transmission to seronegative partners and infants. For example, the current scientific consensus that “undetectable = untransmittable (U = U)” [8] underlies a highly effective safer conception strategy: women living with HIV will not transmit the virus to seronegative partners once they are on treatment and virally suppressed for at least six months [1]. Other lower- and higher-technology safer conception strategies include timing condomless sex to the HIV-positive woman’s fertile days, manual self-insemination with an HIV-negative man’s sperm, pre-exposure prophylaxis (PrEP) to prevent HIV acquisition by seronegative partners, voluntary medical male circumcision, sperm donation, and sperm washing [1, 3, 9, 10].

In the past decade, worldwide, there has been a steady increase in research, service delivery, and advocacy to support women living with HIV to make informed decisions about whether, when, and how to become mothers [1, 3, 11–25]. National normative guidelines on safer conception have been issued in Canada [26], South Africa [9], and the United Kingdom [27], and the World Health Organization delineates strategies for achieving safe and healthy pregnancy in its guidelines on the sexual and reproductive health and rights of women living with HIV [10]. However, HIV service providers often do not feel confident counseling women living with HIV on how they can conceive more safely; the prospect of counseling these women to forgo condoms is foreign and uncomfortable for many clinicians, and some providers hold stigmatizing attitudes about women living with HIV who have children [28–32].

A promising platform for incorporating safer conception counseling is integrated family planning (FP) and HIV service delivery. Since the 1990s, recognizing that both unintended pregnancy and HIV transmission are driven by heterosexual transmission in sub-Saharan Africa, there has been a concerted effort to offer both FP as well as HIV services in integrated service delivery points, either in “one-stop shopping” models, or with facilitated referrals and linkages. Typically, the FP content of integrated service delivery packages focuses on meeting contraceptive need of women affected by HIV, with the aim of reducing unintended pregnancy [33–36]. However, these integrated FP/HIV service delivery settings also may be ideally suited for incorporating preconception counseling and safer conception services, as an essential component of comprehensive FP care. Providers in integrated FP/HIV services are already expected to explore women’s fertility intentions, and, ideally, in light of the aforementioned biomedical advances in the HIV response, these providers will become equally adept at counseling on how to prevent pregnancy as well as how to achieve pregnancy with available safer conception strategies [24, 25].

Furthermore, amidst the nascent policy and programmatic initiatives designed to meet the safer conception needs of women living with HIV, little attention has been paid to the fertility intentions and needs of a particularly vulnerable subpopulation: female sex workers (FSWs) living with HIV. While there is a growing body of evidence of the unmet contraceptive need of FSWs [33, 37–41], a recurring finding in the few studies that address their fertility intentions is that they often aspire to be mothers someday, or desire to have more children in the future [42–44]. In South Africa, among FSWs living with HIV, about half expressed a desire to have children in the future, but their knowledge of safer conception strategies was scant. Although 60% were aware that ART during pregnancy could prevent vertical transmission, just 29% knew that ART use by the seropositive partner could reduce heterosexual transmission during pregnancy attempts, and 4% were aware of PrEP as a safer conception option for the seronegative partner [43].

To inform ongoing country-level and global programs and policies regarding FP/HIV integration and sexual and reproductive health of female key populations, we examined the fertility-related needs and desires of FSWs living with HIV in Tanzania. Tanzania’s adult HIV prevalence is 5.0%, with women bearing a disproportionate burden (6.5% among women compared to 3.5% among men) [45]. FSWs are particularly vulnerable, with a prevalence of 31.4% among women who sell sex in Dar es Salaam [46]. In addition to HIV, these women face substantial reproductive health needs. Unintended pregnancy and abortion is very common, findings from qualitative research conducted among sex workers in southern Tanzania also describe their myriad motivations for wanting to have children, including the desire to earn respect as mothers, avoid stigma of childlessness, and solidify relationships with male partners [44, 47]. Nevertheless, little is known about the experiences of and needs of FSWs regarding safer conception services. Among people living with HIV who do not sell sex, Saleem et al. [31] reported that southern Tanzanian clinicians felt ill equipped to counsel them on how to conceive more safely, despite recognizing their right to have children [31].

### Objectives

Using a service provision assessment methodology, among FSWs living with HIV attending integrated FP/HIV service delivery sites in Dar es Salaam, our specific objectives were to: describe their fertility intentions, need for contraception, and awareness of or interest in safer conception services; and examine the characteristics associated with desire to have a child imminently.

## Materials and methods

### Setting

Study sites were existing Sauti community-based service delivery points in Dar es Salaam. The USAID-supported Sauti project, implemented by Jhpiego, provides comprehensive biomedical services for key populations throughout Kinondoni and Temeke municipalities in Dar es Salaam. Sauti operates at hotspots in the community, which are defined as areas of high HIV prevalence that are mapped and selected with a network of FSW peers, as well on the basis of the routinely reported data on HIV testing yield. The standard delivery package includes, among other services, FP counseling and methods integrated with HIV services. These services include HIV testing and counseling, gender-based violence services, sexually transmitted infection screening and treatment, alcohol and drug screening, and FP counseling and methods. Sauti sites offered condoms, oral contraceptive pills, injectable, and implants on-site. Sauti providers offered referrals for those who sought intrauterine contraceptive devices and sterilization. Biomedical services are offered by trained biomedical providers (i.e., nurses and clinicians), and beneficiaries are mobilized by trained peer educators. According to Sauti standard operating procedures, at every clinical interaction, all FSWs who attend Sauti service delivery sites receive FP screening initiated by the provider. Specifically, Sauti providers are trained to follow a national screening protocol that entails ascertaining whether a female client is likely to be pregnant, and if not, whether she is trying to get pregnant. This line of questioning is followed by questions about current contraceptive use, side effects experienced, and whether she wishes to change her current method (or start a method if not currently using one).

### Data collection activities

The cross-sectional study design consisted of multiple data collection activities at or near selected community-based FP service delivery sites for FSWs living with HIV in Dar es Salaam run by Sauti. All data collection took place between November 2017 and January 2018. These data collection methods were adapted from existing tools for assessing healthcare services, developed by the Population Council [48, 49]. In addition, we drew from MEASURE Service Provision Assessment (SPA) approaches to assessing FP services [50]. Our assessment consisted of client exit interviews (quantitative surveys), structured observations of client-provider interactions, and qualitative interviews with providers and with FSWs living with HIV who wished to conceive. The research protocol and study instruments were approved by two ethical review committees: the institutional review board of the Population Council (New York), and the National Health Research Ethics Committee of the National Institute for Medical Research (Dar es Salaam, Tanzania). Findings from the qualitative data collection activities have been presented previously [51, 52]. This paper presents findings from the quantitative client exit interviews.

#### Client exit interviews

To identify potential participants, Sauti staff reviewed an internal client database and selected a list of women who met the following inclusion criteria: received Sauti HIV testing services in Dar es Salaam in the past year; between the ages of 18 and 49; categorized as FSWs in the Sauti client database (defined as women who reported that they receive money, goods, or favors in exchange for sexual services as a primary source of income [i.e., more than half their monthly income]); and living with HIV, with a date of HIV diagnosis that took place more than one month ago.

This initial list of potential participants was a convenience sample selected by Sauti staff to have roughly the same age distribution of the overall Sauti clientele in Dar es Salaam. Sauti staff then enlisted FSW peer educators to contact and invite 300 eligible women to participate in the study. The target sample size of 300 was calculated to estimate with 6% precision the proportion of participants who aspire to have a child in the next two years, under the conservative assumption of 50% prevalence. The peer educators invited the women to attend a health consultation at one of five Sauti community-based service delivery sites in Kinondoni and Temeke districts in Dar es Salaam. The five study sites were in Bunju, Kawe, and Tandale wards in Kinondoni district; and in Keko and Membweyanga wards in Temeke district. Women were attending services at the invitation of the study team, so they were not necessarily proactively seeking care for a specific concern—whether FP or any other issue—at the time of the consultations.

Upon presenting at the study sites, the potential participant underwent a health consultation with the Sauti provider. Afterwards, an interviewer administered screening questions to ensure that the woman met the inclusion criteria. If the woman was eligible, the interviewer conducted the informed consent process with the potential participant. The interviewer then proceeded to administer a questionnaire on tablets using an open-source electronic data collection software program, KoBo Toolbox (www.kobotoolbox.org).

The survey covered demographic characteristics, reproductive history, contraceptive use, knowledge of “safer conception” strategies, and overall assessment of Sauti services received. To solicit women’s perspectives on service quality, the survey instrument consisted of modules drawn from standardized service provision assessment questionnaires previously developed and validated by the Population Council and MEASURE SPA [48–50]. To understand participants’ awareness of and interest in safer conception strategies, we adapted questions from a study of FSW safer conception knowledge conducted by Rao and colleagues in South Africa [43]. Prior to initiating data collection, the study team translated the survey from English to Kiswahili. To ensure face validity of the survey (i.e., confirming that the questions measure what we intended to measure), we also pre-tested the survey among four FSWs in Dar es Salaam, refining the language to clarify any unclear content.

### Variables and data analysis

The research team conducted descriptive analyses of the exit interview data, calculating simple frequencies to describe the demographic characteristics of participants, as well as their fertility-related needs and knowledge, including awareness of and interest in safer conception strategies. Participants were defined as consistent condom users if they reported that they always used condoms in the past month with both paying clients and nonpaying partners. Use of other, non-condom modern methods (i.e., pill, injectable, intrauterine contraceptive device, implant, or sterilization) was determined based on their responses to a question asking them to report which contraceptive method(s) they used the last time they had sex with a paying partner (since not all women had non-paying partners). Desire for children was defined using the standard demographic definition of fertility desire: wanting to have a child within two years. Participants were classified as virally suppressed if, in response to a question about whether they knew their viral load, they responded “undetectable.” To assess women’s awareness of strategies to get pregnant more safely, we adapted a safer conception survey module that had been used in a previous study among FSWs in South Africa [43]. Specifically, all participants were asked whether they were aware of strategies to get pregnant more safely (and have a safer pregnancy) as women living with HIV. The interviewer read, “For couples in which one or both partners are living with HIV, what ways have you heard of for couples to get pregnant without infecting each other or the baby? For each of the following methods, please tell me if you are aware of each strategy for trying to get pregnant more safely.” This preamble was followed by a brief definition of various biomedical and behavioral strategies for reducing sexual HIV transmission when attempting pregnancy: ART taken by the seropositive partner, PrEP for the seronegative partner, self-insemination with a seronegative man’s sperm, timed condomless sex, sperm washing, sperm donor, and voluntary medical male circumcision. We also asked women to specify whether specific FP content was covered in their consultation, and to report on whether the amount of FP content covered was “too much,” “too little,” or “just right.” We also asked women to report on whether the provider covered specific FP content in their consultations, whether they felt that there was enough FP content covered, and whether they would be interested in learning about safer conception strategies.

We then conducted bivariate analyses to examine correlates of wanting to have a child within the next two years, followed by multivariate logistic regression analysis that accounted for factors that were significantly associated with desire for children in bivariate analyses (at the p<0.05 level), or were documented as strong correlates of fertility desire among women living with HIV in the literature (i.e., number of children, age, relationship status) [53–55]. In addition, to specifically assess whether viral load testing, viral suppression, and awareness of safer conception strategies were associated with fertility desire, we included these variables in the multivariate model. We used the Hosmer-Lemeshow test to assess the regression model’s goodness of fit, and we calculated the model’s variance inflation factor to examine model collinearity.

## Results

Of the 339 women invited by peer educators to participate in the study, 39 were found to be ineligible upon screening by interviewers at study sites. The most common reason for ineligibility was that the woman had never sold sex (n = 34). Other reasons for ineligibility were that the woman was not living with HIV (n = 3), and/or was over age 49 (n = 5). Ineligible women were less likely to be single (41% of ineligibles compared to 55% of eligible, p<0.05), and more likely to have known their HIV status for less than one year (53% of ineligibles versus 33% of eligible, p<0.05). After omitting ineligible women, the exit interview sample consisted of 300 participants.

The median participant age was 32, and 76% had primary education or less. Eleven percent were married or cohabiting, and 73% had at least one nonpaying partner in the past three months. Nearly all (94%) had ever been pregnant, and a large majority (86%) had at least one living child. Nearly one-third (29%) stated that they wished to have a child within two years. Twenty-three percent said that they always used condoms with sexual partners (paying and non-paying) in the past month. In addition, the most frequently mentioned other modern methods that were used were the injectable (21%) and implant (19%). Nearly all were currently on treatment (96%), but far fewer (68%) had received a viral load test in the past six months, and only 13% reported that they had an undetectable viral load (Table 1). Among those who did not wish to get pregnant within two years, 9% were not using condoms consistently, nor were they using another modern method (i.e., unmet need for contraception).

**Table 1:** Exit interview participant characteristics (N = 300).

	n	%
**Age (median, interquartile range)**	32, 12	
**Years selling sex (median, interquartile range)**	4, 5	
**Highest education level attended**		
None/never attended school	27	9
Any primary	202	67
Any secondary	68	23
Any tertiary/(college/university)	3	1
**Marital status**		
Never married/single	164	55
Cohabitating	20	7
Married/living with husband	12	4
Divorced/widowed/separated	104	35
**Had a nonpaying partner in the past 3 months**	218	73
**Ever pregnant**	282	94
**Number of living children**		
0	41	14
1	94	31
2	90	30
3+	75	25
**Desires a child within two years**	86	29
**Always used condoms in the past month**^†^	68	23
**Use of other modern contraception**		
Pill	20	7
Intrauterine system	8	3
Injectable	64	21
Implant	58	19
Female sterilization	4	1
**Unmet need for modern contraception (n = 214)**^††^	20	9
**Currently on ART**	288	96
**Received viral load test in past 6 months**	205	68
**Self-reported undetectable viral load**	39	13

^†^Proportion of women who always used condoms in the past month with paying partners as well as non-paying partners.

^††^Among women who did not wish to have a child within two years (n = 214), unmet need for contraception was defined as the proportion who did not always use condoms in the past month, nor are they using “other modern contraception” (i.e., pill, intrauterine system, injectable, implant, or female sterilization).

**Fig 1:**
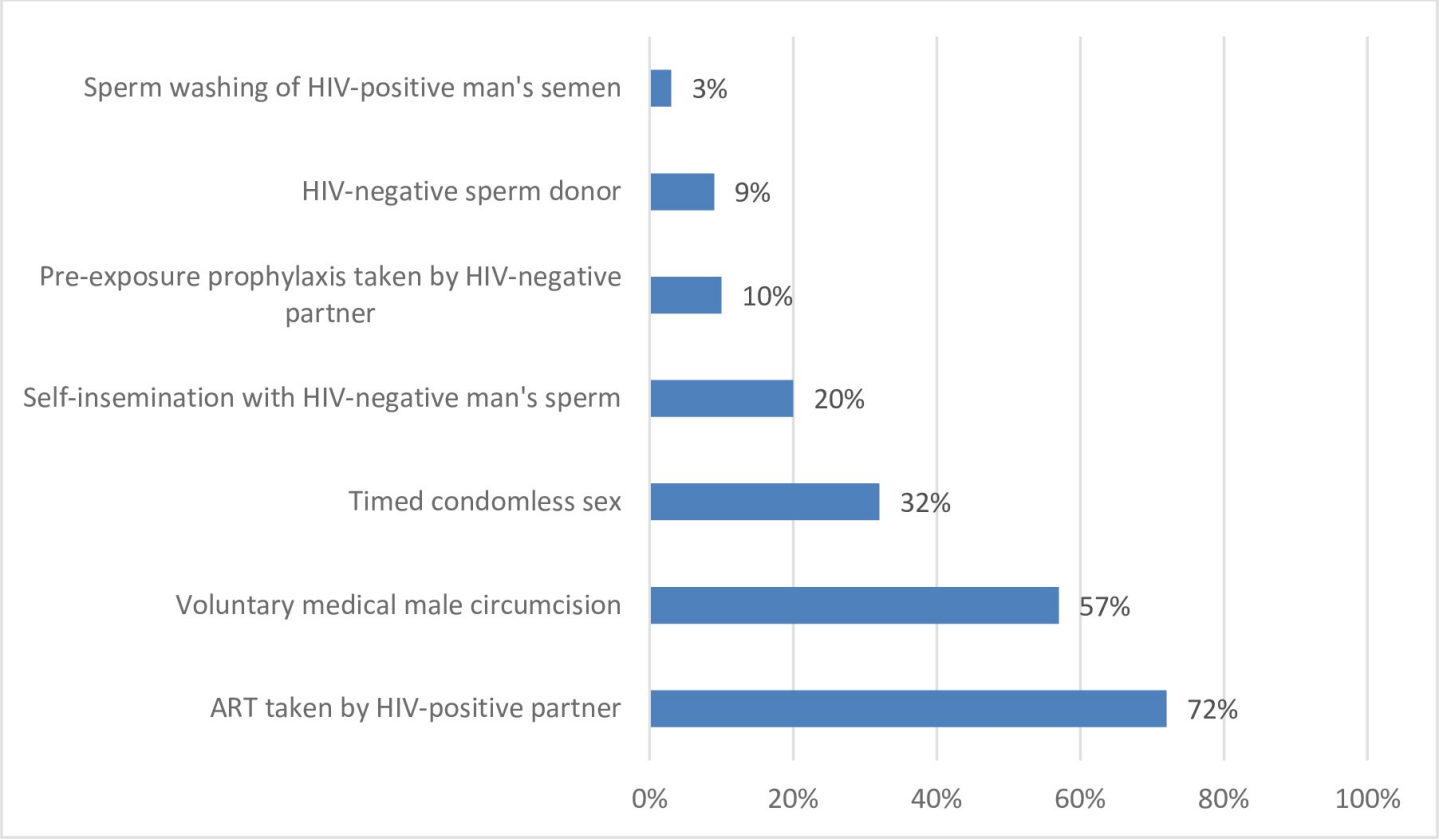
Awareness of safer conception strategies (N = 300).

When asked to specify which strategies they had heard of for people living with HIV to get pregnant more safely, nearly three-quarters said they had heard of having the seropositive partner taking ART. The next most frequently recognized strategies were voluntary medical male circumcision (57%), timed condomless sex (32%), and self-insemination (20%). Far fewer had heard of PrEP (10%), sperm donor (9%), or sperm washing (3%) (Fig 1). Ninety percent recognized at least one of these safer conception strategies.

When asked to reflect on their provider interaction and report whether specific FP content was covered in their consultations, 51% said that the provider asked if she wanted to get pregnant any time in the future, and 42% said they discussed how to get pregnant more safely. One hundred percent reported that the provider treated them respectfully, and 99% would recommend Sauti to their peers. When asked to state how they felt about the amount of FP information provided in the consultation, 9% said there was “too much,” 60% said the amount was “about right,” and 31% stated that the amount of FP information provided was “too little.” (Data not shown)

In bivariate analysis, older women had marginally lower odds of desiring a child within two years (odds ratio [OR] 0.96, 95% confidence interval [CI] 0.93–0.99). Likewise, those who had a greater number of living children had significantly lower odds of wanting a child (OR 0.63, 95% CI 0.50–0.80). Desire for children was positively associated with having had a nonpaying partner in the past three months (OR 2.16, 95% CI 1.15–4.04). In bivariate analysis, desire for children was not significantly associated with education level, having received a viral load test in the past six months, self-reported viral suppression, use of modern contraception, or awareness of at least one safer conception strategy. In multivariate analysis, the only factors that were significantly associated with desire for children were having a nonpaying partner (adjusted odds ratio [AOR] 2.18, 95% CI 1.13–4.20) and number of children (AOR 0.65, 95% CI 0.48–0.87) (Table 2).

**Table 2:** Correlates of wanting a child within two years (N = 300).

	Unadjusted odds ratio, 95% confidence interval	Adjusted odds ratio, 95% confidence interval
Age	0.96, 0.93–0.99*	1.00, 0.96–1.05
Had a nonpaying partner in past 3 months	2.16, 1.15–4.04*	2.18, 1.13–4.20*
Education		
Primary or less	Ref.	Ref.
Secondary or more	1.38, 0.78–2.44	1.16, 0.62–2.15
Number of living children	0.63, 0.50–0.80***	0.65, 0.48–0.87**
Received viral load test in past 6 months	0.66, 0.39–1.11	0.66, 0.36–1.19
Virally suppressed	0.84, 0.39–1.81	1.07, 0.46–2.51
Using modern contraception^†^	0.68, 0.40–1.13	0.65, 0.37–1.15
Aware of at least one safer conception strategy	2.14, 0.79–5.79	2.56, 0.87–7.50
Hosmer-Lemeshow test chi-square		9.98 (p = 0.27)

^†^Always used condoms in the past month, or are using “other modern contraception” (i.e., pill, intrauterine system, injectable, implant, or female sterilization).

*p<0.05

**p<0.01

***p<0.001.

## Discussion

This study corroborates and substantiates the burgeoning research documenting the desire for children among women living with HIV, and the indisputable need and opportunity to provide explicit safer conception counseling and services to these women [1–7]. At the same time, among women who did not want to get pregnant within the next two years, 10% were neither using condoms consistently, nor using another modern contraceptive method. We identified one other study that estimated unmet contraceptive need among FSWs living with HIV, in which Long and colleagues reported 64% unmet need for “non-barrier modern contraception” among Kenyan FSWs living with HIV [56]. However, this substantially higher estimate is not comparable to our estimate, which assessed need for contraception defined more broadly; we did not limit our assessment to need for “non-barrier” contraception. Respected, trusted integrated services such as Sauti’s are an ideal platform in which to ensure the FP conversation is broadened to not only address women’s contraceptive needs, but also provide nonjudgmental community-based services in which to support their desire to have children. The finding that nearly one-third of exit interview participants felt that the FP content covered in their consultation was “too little” speaks to their interest in learning more about these issues. At minimum, a FP discussion presents a teachable opportunity to impress upon HIV-positive women that there are effective biomedical and behavioral strategies that can, in some cases, virtually eliminate the possibility of transmitting HIV to a partner or to an infant. Some of these strategies—such as sperm washing—are not widely available for most HIV-positive women in Tanzania [57], but ART adherence and timed condomless sex are arguably readily accessible as safer conception options. The finding that those who wanted children imminently were no more likely to report viral suppression is concerning. One would hope that women living with HIV who are contemplating pregnancy would be aware of and supported to achieve viral suppression to minimize risk of HIV transmission to a seronegative partner. Furthermore, informing HIV-positive women of the possibility of having a safe pregnancy has the potential to motivate her to remain on treatment and seek timely antenatal care.

Study participants were not only women living with HIV, but they were also women who sell sex. Due to both internal and external stigma, both women and providers alike may believe that HIV-positive FSWs are inherently uninterested in (or unfit to) have children, but our study findings demonstrate that a sizable proportion of these women aspire to become mothers, or complete their families (among those who already are mothers). Notably, Cernigliaro and colleagues [58] reported that, among FSWs in the Dominican Republic, internalized stigma was associated with greater fertility desire; becoming a mother may provide these marginalized women with a sense of self-worth and acceptance [58]. The finding that those with nonpaying partners have a higher odds of wanting children is intuitive, since these nonpaying partners often are men with whom FSWs have more intimate relationships than with paying clients. Findings from previous research supports this hypothesis; some FSWs report less consistent condom use with nonpaying partners because they feel that condoms compromise the intimacy of the sexual relationship, or they aspire to get pregnant with those partners [59, 60]. As articulated cogently by Ippoliti and colleagues (2017), female key populations such as FSWs have a host of sexual and reproductive health needs, among which safer conception services often are neglected [61].

Results should be interpreted with the study context and limitations in mind. Sauti community-based sites offer a range of services beyond FP, and study participants were not necessarily proactively seeking FP counseling. In addition, to contextualize the low reported prevalence of recent viral load testing and self-reported viral suppression, it is worth underscoring that women are recommended to receive viral load testing within six months of treatment initiation, and once a year thereafter. The low observed levels of viral load testing and self-reported viral suppression may partly be due to the fact that many women were not “due” to undergo viral load testing in the past six months. In addition, viral load testing may not always be available due to, for example, reagent stockouts or insufficient maintenance of viral load testing machines. Nevertheless, the finding that there was no significant correlation between fertility desire and either of these measures remains concerning. Moving forward, providers would benefit from guidance about the minimum FP screening and counseling that they should offer at every clinical interaction with HIV-positive women, with explicit consideration of how to address the possibility of having children in the future.

Comprehensive contraceptive counseling must remain in place as a foundation of integrated FP/HIV services for female key populations, coupled with a full range of modern methods available on-site or through referral. In addition, however, there is a need to ensure that services are prepared to address HIV-positive women’s desire to have children in the future. Policymakers and service delivery organizations in Tanzania and elsewhere can consider building on existing national guidelines from South Africa, consensus statements from safer conception researchers and advocates for people living with HIV (PLHIV), and a safer conception toolkit that has been tested in Kenya, for instance [1, 9, 17]. In addition, once PrEP becomes available more widely, this may present an opportunity to test the acceptability of pre-conception PrEP for seronegative partners in mixed-status relationships.

Adolescent girls and young women remain disproportionately burdened by HIV in sub-Saharan Africa, and as they age into their reproductive years, the question of whether and how to have children will become increasingly salient. As demonstrated by our findings, FSWs are no exception to this reality. The growing consensus among researchers and advocates alike is that there is an urgent need for policies and programs to help HIV-positive women achieve pregnancy, while minimizing HIV risk [1].

Furthermore, as the international HIV response continues to evolve in the U = U context, our study builds the evidence base to support all women living with HIV to plan whether and when to have children, while minimizing risk of onward HIV transmission to partners and infants.

## Supporting information

S1 File Client exit interview survey instrument–English. (DOCX)

S2 File Client exit interview survey instrument–Kiswahili. (DOCX)
