# Supplementary material for: Safer conception for female sex workers living with HIV in Dar es Salaam, Tanzania: Cross-sectional analysis of needs and opportunities in integrated family planning/HIV services
Source: PLoS One. 2020 Jul 21;15(7):e0235739. doi: 10.1371/journal.pone.0235739 (PMC7373272; doi:10.1371/journal.pone.0235739)
Supplement: S1 File — (DOCX) [file pone.0235739.s001.docx]

# Safer conception for female sex workers living with HIV in Dar es Salaam, Tanzania: cross-sectional analysis of needs and opportunities in integrated family planning/HIV services

# Client exit interview survey instrument - English

| **No** | **Questions** | **Coding** |
| --- | --- | --- |
| 00 | Interview start time | Hr:[­­­­­___] Min:[____] **24 hours** |
| 01 | Interview Date | _____ / _____ / _____  *(Day/ Month/ Year)* |
| 02 | Sauti site  ***[Write in name of site.]*** | ________________________ |
| 03 | Interviewer Code  ***Enter the code of the interviewer*** | [___\|___] |
| 04 | Participant Identification Number (PID) | \|  \|  \|  \| \| --- \| --- \| --- \| |

**SECTION 1: BACKGROUND CHARACTERISTICS & ELIGIBILITY SCREENING**

***In this section, I am going to ask you some general questions about your background and socioeconomic status. Remember that your answers are confidential.***

***Please circle the appropriate response***

| **No.** | **Questions and filters** | **Coding categories** | | **Codes** | | **Skip to** | | **Training notes** |
| --- | --- | --- | --- | --- | --- | --- | --- | --- |
|  | How old were you at your last birthday? If you are not sure, can you give your best estimate?  ***[Try to help her estimate her age.]*** | Age in complete years  Don’t know | | ______  99 | | Ineligible if <18 years | | Eligible women must be 18+. |
|  | What district do you live in? | Kinondoni  Temeke  Ilala  Ubungo  Kigamboni  Other (Specify) | | 1  2  3  4  5  96 | |  | |  |
|  | What ward do you live in? | Bunju  Azimio  Buza  Hanasifu  Kigamboni  Msasani  Mtoni  Mwananyamala  Tandale  Tandika  Kurasini  Yombo  Other (Specify)  ___________________ | | 1  2  3  4  5  6  7  8  9  10  11  12  96 | |  | |  |
|  | What is the highest level of school you attended: primary, secondary, or higher? | None/never attended school  Primary  Secondary  Any tertiary (college/university) | | 1  2  3  4 | |  | |  |
|  | What is your current marital status? | Never married/single  Cohabitating  Married/living with husband  Divorced/widowed/separated  No response | | 1  2  3  4  99 | |  | |  |
|  | How long have you been selling sex?  ***[Ask her how many years. If less than one year, indicate “1” for number of years.]***  ***Please take your time and try to give us your best estimate.*** | Has never sold sex  Years | | 0  _____ | | 🡪Ineligible | | This is an eligibility screening question. If the response is “Has never sold sex,” the participant is not eligible. |
|  | Thinking of the past three months, would you say that your main source of income has been selling sex? | No  Yes | | 0  1 | |  | |  |
|  | Are you currently living with HIV? | No  Yes | | 0  1 | | Q110 | | 🡪Ineligible |
|  | How long ago did you first learn that you were living with HIV? | <1 month  1-11 months  1-4 years  >5 years | | 0  1  2  3 | |  | | 🡪Ineligible |
|  | ***[Interviewer: Indicate whether or not she is eligible based on her responses to Q101, 104, 105, 106, and 107.]*** | Ineligible  Eligible | | 0  1 | | END | |  |
| **CONSENT [Only Eligible Participants]**  READ: I am going to read you a document that explains this study. Please stop me at any point and ask questions if anything is unclear. When I finish reading, I am going to ask you to sign this form to indicate that you understand the study and agree to participate in this survey.  [READ CONSENT FORM AND CONDUCT CONSENT PROCESS] | | | | | | | | |
| Q111 | Are you willing to participate in the interview? | No  Yes | 0  1 | | 🡪Ineligible | |  | |

**SECTION 2: REPRODUCTIVE HISTORY**

| **No.** | **Questions and filters** | **Coding categories** | **Codes** | **Skip to** |
| --- | --- | --- | --- | --- |
|  | Have you ever been pregnant, even if no child was born? | No  Yes | 0  1 | Q301a |
|  | Do you have any living children to whom you gave birth? | No  Yes | 0  1 | Q204 |
|  | How many living children do you have, to whom you gave birth? | Number of children | ____ |  |
|  | With how many different men have you gotten pregnant? | One (i.e., all pregnancies with same man)  Two  Three or more  Don’t know | 1  2  3  99 |  |
|  | Have you ever had a pregnancy that did not end in a live birth? | No  Yes  Don’t know | 0  1  99 | Q301a |
|  | Of all of your pregnancies that did not end in a live birth, how many times have you experienced each of the following outcomes?  ***[Read each of these birth outcomes aloud and ask her to state how many miscarriages, stillbirths, and induced abortions that she has experienced. Enter “0” for each outcome that she has not experienced.]*** | Miscarriage  Stillbirth  Induced abortion | __  __  __ |  |
|  | ***[Only ask this question if she reports having had an induced abortion in Q206.]***  From which of the following places or providers have you received abortion care or abortion medication? I’m interested in any care you received to induce abortion.  **SELECT ALL THAT APPLY.** | Public health facility  NGO health facility  Private commercial health facility  Pharmacist  Other non-health professional (e.g., friends, relatives, etc.)  Other (*SPECIFY*): _______________ | 1  2  3  4  96 |  |

**SECTION 3: SEXUAL behavior AND FAMILY PLANNING**

***Next, I will ask you questions about issues related to your sexual health and sexual behavior.***

***Please remember that everything you say to me is confidential.***

***For the purpose of this interview,* “Having sex” *means having vaginal sex only.***

| **No.** | **Question** | **Coding categories** | **Codes** | **Skip to** |
| --- | --- | --- | --- | --- |
| Q301a | In the time since you received your HIV diagnosis, have you ever received counselling about how to prevent pregnancy (i.e., family planning)? | Yes  No  Don’t know | 1  0  98 | 🡪Q302  🡪Q302 |
| Q301b | Where did you receive this counseling on preventing pregnancy?  **SELECT ALL THAT APPLY.** | Government health facility  Private health facility  Private pharmacy  Other shop  Community health worker  Friend/relative  Sauti services  Another NGO  Other (specify):  ­­­­­­­­­­­­­­­­­­­­____________________ | 1  2  3  4  5  6  7  8  96 |  |
| Q302 | Think of the last time you had sex with a paying client. At that time, which of the following pregnancy prevention methods did you use? Please tell me all the methods you used at that time to prevent pregnancy, HIV, or STIs.  *(If respondent only mentions condoms, you would tick “condoms” accordingly, but in addition, probe with,* ***“In addition to condoms, were you using any other contraceptive method at that time?”*** *Mark all that apply.)* | Pill  Intrauterine device (IUD)  Injectable  Implant  Male condom  Female condom  Female sterilization  Male sterilization  Lactational amenorrhea method (LAM)  Rhythm (Calendar) method  Withdrawal  No method  Other (specify):  ________________________ | 1  2  3  4  5  6  7  8  9  10  11  0  96 |  |
| Q302a | Now thinking of the past month, how consistently do you feel you were able to use condoms with all of your paying clients?  ***[Read response options and tick her answer.]*** | Never  Rarely  Most of the time  Always | 0  1  2  3 |  |
| ***Now, I am going to talk to you about non-paying sexual partners, which could be a spouse, cohabiting partner or boyfriend. It could also be a sexual partner where you only had a single sexual encounter or several sexual encounters over a short period of time without the expectation of a relationship.*** | | | | |
| Q303 | During the past three (3) months, how many non-paying sex partners did you have in total?  [Ask the woman to give an estimate] | None  Total  No response | 0  ______  99 | 🡪Q305  🡪Q305  🡪Q305 |
| Q304 | Think of the last time you had sex with a non-paying partner. At that time, which pregnancy prevention methods did you use? Please tell me all the methods you used at that time.  *(If respondent only mentions condoms, you would tick “condoms” accordingly, but in addition, probe with,* ***“In addition to condoms, were you using any other contraceptive method at that time?”*** *Mark all that apply.)* | Pill  Intrauterine device (IUD)  Injectable  Implant  Male condom  Female condom  Female sterilization  Male sterilization  Lactational amenorrhea method (LAM)  Rhythm (calendar) method  Withdrawal  No method  Other (specify):  ________________________ | 1  2  3  4  5  6  7  8  9  10  11  0  96 |  |
| Q304a | Now thinking of the past month, how consistently do you feel you were able to use condoms with all of your non-paying partners?  ***[Read response options and tick her answer.]*** | Never  Rarely  Most of the time  Always | 0  1  2  3 |  |
| Q305 | ***[If using pill/IUD/injectable/implant/sterilization]*** Where did you most recently obtain the method, e.g. pill/IUD/injectable/implant/sterilization?  **PROMPT RESPONDENT BY READING THE LIST HERE.** | HIV testing site  HIV care and treatment site  Another HIV services  MNCH/FP service delivery site  Other health care delivery site  Sauti community-based service  Pharmacy  Other shop  Community health worker  Friend/relative  Other (specify):  ­­­­­­­­­­­­­­­­­­­­_________________________ | 1  2  3  4  5  6  7  8  9  10  96  🡪Q401  🡪Q401 |  |
| Q305a | Was the facility or service public, private, or NGO sector?  ***[Private sector refers to a commercial or for-profit facility, in contrast to a non-profit NGO service.]*** | Public sector  Private commercial sector  NGO  Don’t know | 0  1  2  98 |  |
| Q305b | Can you tell us the name of the facility or program? | No  Name: ________________________ | 0  1 |  |
| Q306 | ***[If said only using condom, OR if they say they are not using any method at all.]***  There are effective contraceptive methods, such as the pill, injectable, implant, and sterilization. Can you tell me why you choose not to use any of these effective methods?  *(You do not need to read the response options, but probe with statements like, “Is there any reason?” Record all the responses mentioned by participant.)* | ***Fertility-related reasons***  She has infrequent sex  Menopausal/hysterectomy  She can’t get pregnant  Has not menstruated since last birth  Breastfeeding  Up to God/fatalistic  Wants to get pregnant  ***Opposition to use***  She is opposed to these methods  Partner(s) is/are opposed  Others are opposed  Religious prohibition  ***Lack of knowledge***  Does not know these methods  Does not know where to get methods  ***Method-related reasons***  Side effects/health concerns  Lack of access/too far  Costs too much  Preferred method not available  No such methods available  Inconvenient to use  Interferes with body’s normal processes  Other (specify):  ­­­­_________________________ | 1  2  3  4  5  6  7  8  9  10  11  12  13  14  15  16  17  18  19  20  96 |  |

**SECTION 4: FERTILITY DESIRE AND SAFER CONCEPTION**

| **No.** | **Question** | **Coding categories** | **Codes** | **Skip to** |
| --- | --- | --- | --- | --- |
| Q401 | Would you like to have a (or another) child someday, or would you prefer not to have any (more) children? | Have a/nother child  No more/none  Says she cannot get pregnant  Don’t know | 1  2  3  98 | 🡪Q403  🡪Q404a  🡪Q403 |
| Q401a | How long would you like to wait from now before the birth of a/another child? | Currently trying to get pregnant  Within one year  Within two years  More than two years from now  Don’t know | 0  1  2  3  98 | SW403  SW403 |
| Q402 | [If trying to get pregnant within the next two years]  With whom are you trying to have a child? | Current husband/boyfriend  Ex-husband/ex-boyfriend  Client  Other (specify):  ________________ | 1  2  3  96 |  |
| Q403 | If you got pregnant now, how would you feel? Would you be very upset, a little upset, a little pleased, or very pleased? | Very upset  A little upset  Neither upset nor pleased  A little pleased  Very pleased | 1  2  3  4  5 |  |
|  | Many people who think about having children are affected by HIV. For couples in which one or both partners are living with HIV, what ways have you heard of for couples to get pregnant without infecting each other or the baby? For each of the following methods, please tell me if you are aware of each strategy for trying to get pregnant more safely:  ***[Read each item and note whether she is aware or unaware of each method.]*** | | | |
| Q404a | HIV medications taken by the HIV-positive partner . This will reduce risk of passing HIV from the positive to the negative partner. | Aware  Unaware  Don’t know | 1  0  98 |  |
| Q404b | HIV medications taken by the HIV-negative partner (i.e., pre-exposure prophylaxis or PrEP). This will reduce risk of having the negative partner acquire HIV from the positive partner. | Aware  Unaware  Don’t know | 1  0  98 |  |
| Q404c | HIV medications taken by the HIV-positive woman during pregnancy. This reduces the risk of having the woman pass HIV on to her infant. | Aware  Unaware  Don’t know | 1  0  98 |  |
| Q404d | Self-insemination. This is when the HIV-negative man ejaculates into a condom or container and then manually deposits semen into the HIV-positive woman’s vagina. This reduces the risk of having the positive woman pass the virus to the negative man. | Aware  Unaware  Don’t know | 1  0  98 |  |
| Q404e | Timed unprotected intercourse. This is when couples only have unprotected sex during the few days a month when the woman is most fertile. This reduces the risk of having the positive partner pass the virus to the negative partner. | Aware  Unaware  Don’t know | 1  0  98 |  |
| Q404f | Sperm washing. This is technology to cleanse an HIV-positive man’s sperm of HIV. This reduces the risk of the positive man passing the virus to the negative woman. | Aware  Unaware  Don’t know | 1  0  98 |  |
| Q404g | Sperm donor. This refers to having an HIV-negative man (who is not the male partner) donate sperm to impregnate the woman. If either partner is HIV-positive, using sperm of a “third-party” HIV-negative sperm donor instead of having unprotected intercourse reduces risk of passing the virus between the serodiscordant couple. | Aware  Unaware  Don’t know | 1  0  98 |  |
| Q404h | Medical male circumcision. This is the surgical removal of skin from the HIV-negative man’s penis. This reduces the risk of a positive woman passing the virus to her negative partner. | Aware  Unaware  Don’t know | 1  0  98 |  |
| Q405 | How interested are you in learning about how HIV-positive women can try to get pregnant without infecting the partner or the baby? | Not at all interested  A little interested  Very interested | 0  1  2 |  |
| Q406 | Has a healthcare provider ever provided you counseling or services about how HIV-positive women can make conception and pregnancy safer? | Yes  No  Don’t remember | 1  0  98 |  |

**SECTION 5: ASSESSMENT OF FP SERVICES**

***Now I’m going to ask you about your TODAY’s consultation with the Sauti service provider. These questions I will ask you are in reference to your TODAYs interaction and counseling with him/her only, not from other interactions you may have had in the past with other providers.***

| **No** | **Questions and filters** | **Coding categories** | **Codes** | **Skip to** |
| --- | --- | --- | --- | --- |
| Q501 | Did the provider ask you if you are currently trying to get pregnant? | Yes  No  Don’t know | 1  0  98 |  |
| Q502 | Did the provider ask you if you wish to get pregnant at any time in the future? | Yes  No  Don’t know | 1  0  98 |  |
| Q503 | Did the provider discuss with you how to get pregnant while minimizing the risk of passing on the HIV virus to your partner or your baby? | No  Yes  Don’t know | 0  1  98 |  |
| Q504 | Did the provider discuss with you the effect that HIV can have on your fertility (i.e., HIV reduces fertility)? | Yes  No  Don’t know | 1  0  98 |  |
| Q505 | Prior to today’s consultation, had you ever talked with any Sauti provider about how to get pregnant more safely (i.e. getting pregnant while minizing the risk of HIV transmission to your partner or unborn baby)? | Yes  No  Don’t know | 1  0  98 |  |
| Q506 | Did the provider ask you if you are currently using any method to prevent pregnancy? | Yes  No  Don’t know | 1  0  98 |  |
| Q507 | Some women may use different methods of preventing pregnancy with different partners. For example, they may be using condoms to prevent pregnancy with clients, but they may not use condoms with their boyfriends. Did the provider ask you about whether your contraceptive use is different with different partners? | Yes  No  Don’t know | 1  0  98 |  |
| Q508 | Did the provider ask you if you were currently using any method to prevent pregnancy with your non-paying clients? | Yes  No  Don’t know | 1  0  98 |  |
| Q509 | Did the provider ask you if you were having any problems with the pregnancy prevention method(s) you are using? | Yes  No  Don’t know | 1  0  98 |  |
| Q510 | Did the provider ask you if you had a preference for any particular pregnancy prevention method? | Yes  No  Don’t know | 1  0  98 |  |
| Q511 | Which of the following methods did the provider discuss with you?  ***READ ANSWERS ALOUD AND* *SELECT ALL THAT APPLY.*** | Pill  Intrauterine device (IUD)  Injectable  Implant  Male condom  Female condom  Female sterilization  Male sterilization  Lactational amenorrhea method  Rhythm method  Withdrawal  Emergency contraception  No method discussed  Other (*SPECIFY*):  ________________________ | 1  2  3  4  5  6  7  8  9  10  11  12  0  96 |  |
| Q512 | Did the provider mention side effects of different methods with you? | Yes  No  Don’t know | 1  0  98 |  |
| Q513 | Did the provider discuss how antiretroviral medications may affect how well contraceptive methods work? | Yes  No  Don’t know | 1  0  98 |  |
| Q514 | Did you receive a contraceptive method today? | No, and I do not wish to use a method.  Yes  I already had a method | 0  1  2 | Q517  Q517 |
| Q515 | Were you given a prescription or referral for a method today? | No  Yes, prescribed a method  Yes, referred for a method | 0  1  2 | Q517 |
| Q516 | Which method(s) did you receive or were given a prescription or referral?  ***(Select all that apply.)*** | Pill  Intrauterine device (IUD)  Injectable  Implant  Male condom  Female condom  Female sterilization  Male sterilization  Lactational amenorrhea method  Rhythm method  Withdrawal  Emergency contraception  No method discussed  Other (*SPECIFY*):  ________________________ | 1  2  3  4  5  6  7  8  9  10  11  12  0  96 |  |
| Q517 | Did the provider talk to you about telling your partner(s) about your HIV status (i.e., disclosure)? | Yes  No  Don’t know | 1  0  98 |  |

**SECTION 6. HIV-RELATED BEHAVIORS**

| Q601 | Are you currently receiving services at an HIV care and treatment center? | No  Yes | 0  1 | 🡪Q603 |
| --- | --- | --- | --- | --- |
| Q601a | What are the main reasons you have not registered in care?  ***SELECT ALL THAT APPLY*** | Feel healthy/not sick  Stigma, don't want others to know  Cost/can’t afford to go to clinic  Distance to health facility is too far  Waiting time or hours at clinic not good  Poor attitude of health care workers  I don’t know where to register  Other______________  Don't Know | 1  2  3  4  5  6  7  96  98 | All Q701 |
| Q602 | Have you tested for viral load during the past 6 months? | Yes  No | 1  0 | 🡪Q603 |
| Q602a | Do you remember the range of your viral load results? | undetectable  detectable  Have not received results yet  Don’t remember | 0  1  2  98 |  |
| Q603 | Are you currently taking antiretroviral therapy? | No  Yes  Don’t know | 0  1  98 | 🡪Q605 |
| Q604 | During the past 7 days, on how many days have you missed all your pills? | No days  One day  Two days  Three days  More than three days | 0  1  2  3  4 |  |
| Q605 | How often do you see your HIV doctor? | Every 1 month  Every 2-3 months  Every 4-6 months  Other_________ | 1  2  3  96 |  |
| Q606 | Besides your HIV care providers (doctors/ nurses), have you told anyone that you are living with HIV? | Yes  No | 1  0 | 🡪Q701 |
| Q607 | If yes, who did you tell?  SELECT ALL THAT APPLY | Paying clients  Casual non-paying partners  Boyfriend/Husband  Parents  Siblings  Support group members  Peer educators  Friends  Children  Other (Specify)__________ | 1  2  3  4  5  6  7  8  9  96 |  |

**SECTION 7. CLIENT SATISFACTION**

| Q701 | Did you feel that the family planning information given to you during your consultation today was too little, too much, or just about right? | Too little  Too much  About right | 1  2  3 |  |
| --- | --- | --- | --- | --- |
| Q702 | Did you feel that you had privacy during your consultation with the provider? In other words, did you feel comfortable that others could not see or hear you? | No  Yes | 0  1 |  |
| Q703 | Did you feel that the provider treated you respectfully? | Yes  No | 1  0 |  |
| Q704 | Did the provider give you the opportunity to ask questions? | No  Yes | 0  1 |  |
| Q705 | Would you recommend Sauti services to other women like you? | No  Yes | 0  1 | Q707 |
| Q706 | Why wouldn’t you recommend Sauti services to other women? |  |  |  |
| Q707 | Result of the interview | Complete  Incomplete | 1  2 |  |
| Q708 | If incomplete, what was the reason? | __________________________ |  |  |
| Q709 | Interview end time | Hr:[___] Min:[____] |  | 24 hour |

***Thank you for your time.***
