# Supplementary material for: Safer conception for female sex workers living with HIV in Dar es Salaam, Tanzania: Cross-sectional analysis of needs and opportunities in integrated family planning/HIV services
Source: PLoS One. 2020 Jul 21;15(7):e0235739. doi: 10.1371/journal.pone.0235739 (PMC7373272; doi:10.1371/journal.pone.0235739)
Supplement: S2 File — (DOCX) [file pone.0235739.s002.docx]

# Safer conception for female sex workers living with HIV in Dar es Salaam, Tanzania: cross-sectional analysis of needs and opportunities in integrated family planning/HIV services

# Client exit interview survey instrument - Kiswahili

| **Na.** | **Maswali** | **Alama** |
| --- | --- | --- |
| 01 | Tarehe ya Mahojiano | _____ / _____ / _____  *(Siku/ Mwezi/ Mwaka)* |
| 02a | Mkoa | [___] |
| 02b | Wilaya | [___] |
| 02c | Kata | [___] |
| 03 | Alama ya Anayehoji  ***Ingiza alama ya Anayehoji*** | [___\|___] |
| 04 | Namba Ya Utambulisho Ya Mshiriki | \|  \|  \|  \| \| --- \| --- \| --- \| |
| 05 | Muda wa Kuanza Mpaka Kumaliza Mahojiano  ***Tafadhali ingiza makadirio ya muda wa mahojiano kulingana na muda wa kuanza na wa kumaliza*** | 05.a. Muda wa kuanza:__________  05.b: Muda wa kumaliza:___________ |
| 06 | Matokeo ya mahojiano | 1. Yalimalizika 2. Hayakumalizika   *Kama hayakumalizika, sababu zilikuwa ni zipi:___________________* |

**SEHEMU 1: TAARIFA ZA AWALI**

***Katika kipengele hiki, nitaenda kukuuliza maswali ya kawaida kuhusiana na historia yako na hali yako ya kiuchuminakijamii. Kumbuka kwamba majibu yako yatawekwa kuwa usiri. Tafadhali zungushia jibu lililo bora zaidi.***

| **Namba.** | **Maswali na vikaguzi** | **Ngazi za Alama** | **Alama** | **Rejea** |
| --- | --- | --- | --- | --- |
| SW101 | Ulikuwa na umri gani siku yako ya mwisho ya kuzaliwa? | Umri kwa miaka yote    Sijui | ____  99 |  |
| SW102 | Ni kiwango gani kikubwa cha shule ulichohudhuria: msingi, sekondari, au juu zaidi? | Hakuna/Sijawahi kuhudhuria shule  Sijamaliza shule ya msingi  Sijamaliza shule ya sekondari  Kiwango chochote cha juu zaidi (hajamaliza chuo/chuo kikuu) | 1  2  3  4 |  |
| SW103 | Hali yako ya sasa ya ndoa ikoje? | Sijawahi kuolewa/niko mwenyewe  Sijawahi kuolewa/tunaishi pamoja bila ndoa  Nimeolewa/ninaishi na mume  Nimetalikiwa/mjane/tumeachana  Hakuna jibu | 1  2  3  4  99 |  |
| SW104 | Ukifikiria miezi mitatu iliyopita, unaweza kusema kwamba chanzo chako kikubwa cha kipato kimekuwa ni kuuza ngono? | Hapana  Ndiyo | 0  1 | 🡪MWISHO |
| SW105 | Ulikuwa una umri gani ulipoanza kuuza ngono?  ***Tafadhali tumia muda wa kutosha na ujaribu kutupa makadirio sahihi zaidi.*** | Umri kwa miaka kamili  Sijui | ______  99 |  |

**SEHEMU 2: HISTORIA YA UZAZI**

| **Na.** | **Maswali na vikaguzi** | **Ngazi za alama** | **Alama** | **Rejea** |
| --- | --- | --- | --- | --- |
| SW201 | Ulishawahi kuwa mjamzito, hata kama hakuna mtoto aliyezaliwa? | Hapana  Ndiyo | 0  1 | SW301a |
| SW202 | Una mtoto yeyote anayeishi ambaye ulimzaa? | Hapana  Ndiyo | 0  1 | SW205 |
| SW203 | Una watoto wangapi uliowazaa, ambao wako hai? | Idadi ya watoto | ____ |  |
| SW204 | Umezaa watoto na wanaume tofauti wangapi? | Mmoja (i.e.,wote ni wa baba mmoja)  Wawili  Watatu au zaidi  Sijui | 1  2  3  99 |  |
| SW205 | Ulishawahi kuwa na mimba ambayo haikuishia kuwa na mtoto aliye hai? | Hapana  Ndiyo  Sijui | 0  1  99 | SW301 |
| SW206 | Katika mimba zako zote ambazo hazikuishia kuwa na mtoto aliye hai, ni mara ngapi umepata matokeo yafuatayo? | Mimba kuharibika  Kuzaa mtoto alikwisha kufa  Kutoa mimba kwa kukusudia  Mengine (*TAJA*): ______________ | __  __  __  96 |  |
| SW207 | [Uliza tu swali hili kama atajibu kuwa amewahi kutoa mimba kwa kukusudia katika SW206]  Ni sehemu zipi kati ya hizi au vituo vya kutolea huduma ambako umepata msaada wa kutolewa mimba au dawa za kutolea mimba? Ninataka kufahamu huduma yoyote uliyoipokea ya kutoa mimba pamoja na huduma yoyote uliyopata baada ya kutoa mimba. | Kituo cha afya cha umma  Kituo cha afya cha shirika lisilo la kiserikali  Hospitali binafsi ya kibiashara  Mkunga wa jadi  Mfamasia  Mengineyo *(TAJA):* _______________ | 1  2  3  4  5  96 |  |

**SEHEMU 3: TABIA ZA NGONO NA UZAZI WA MPANGO**

***Sasa, nitakukuuliza maswali kuhusu masuala yanayohusiana na afya yako ya uzazi na tabia ya ngono.***

***Tafadhali kumbuka kwamba kila kitu unachoniambia kitawekwa kwa siri.***

***Kwa madhumuni ya mahojiano haya, “Kufanya ngono” maana yake ni kufanya mapenzi kupitia ukeni, isipokuwa kama imeelezwa vinginevyo***.

| **Na.** | **Swali** | **Ngazi za alama** | **Alama** | **Rejea** |
| --- | --- | --- | --- | --- |
| SW301a | Tangu kipindi ulichopata majibu ya kipimo chako cha VVU, ulishawahi kupokea ushauri juu ya namna ya kuzuia mimba (nikimaanisha ushari juu ya uzazi wa mpango)? | Ndiyo  Hapana  Sijui | 1  0  98 | 🡪SW302  🡪SW302 |
| SW301b | Ulipokelea wapi ushauri huu juu ya kuzuia kupata mimba? | Kituo cha afya cha serikali  Kituo binafsi cha afya  Famasi binafsi  Duka lingine  Mfanyakazi wa afya wa jamii  Rafiki/Ndugu  Mengineyo (Elezea)  ­­­­­­­­­­­­­­­­­­­­_________________________ | 1  2  3  4  5  6  96 |  |
| SW302 | Fikiri mara ya mwisho kufanya ngono na mteja anayelipia. Wakati huo, ni njia zipi za kuzuia mimba kati ya hizi ambazo ulitumia? Tafadhali niambie njia zote ulizotumia wakati huo.  *(Kama mhojiwa ametaja kondom tu, chunguza kuwa,* ***“Pamoja na kondom, ulikuwa unatumia njia nyingine yoyote ya kuzuia ujauzito wakati ule?”*** *Wekea alama yote yaliyo sahihi)* | Kidonge  Kitanzi  Sindano  Kipandikizi  Kondomu ya kiume  Kondomu ya kike  Kufunga uzazi kwa mwanamke  Kufunga uzazi kwa mwanaume  Njia ya asili ya uzazi wa mpango ya unyonyeshaji au  Kumwaga mbegu za kiume nje  Hakuna njia yoyote  Nyingineyo (Elezea):  ________________________ | 1  2  3  4  5  6  7  8  9  10  11  0  96 |  |
| ***Sasa, nitaenda kuzungumza na wewe kuhusiana na marafiki wa ngono wasio wa malipo, ambaye anaweza kuwa ni mwenzi, mtu unayeishi naye au rafiki wa kiume. Inaweza pia ikawa ni rafiki yako wa kimapenzi ambapo ulifanya nae tendo la ngono mara moja au mara kadhaa kwa kipindi cha muda mfupi bila mategemeo ya kuwa na uhusiano.*** | | | | |
| SW303 | Katika kipindi cha miezi mitatu (3) iliyopita, umekuwa na marafiki wangapi wasio wa malipo kwa ujumla? | Hakuna  Jumla  Sikumbuki  Hakuna jibu | 0  ______  98  99 | 🡪SW304a |
| SW304 | Fikiri juu ya mara ya mwisho kufanya ngono na rafiki asiye wa malipo. Wakati huo, ni njia zipi za kuzuia mimba kati ya hizi ambazo ulitumia? Tafadhali niambie njia zote ulizotumia wakati huo.  *(Kama mhojiwa ametaja kondom tu, chunguza kuwa,* ***“Pamoja na kondom, ulikuwa unatumia njia nyingine yoyote ya kuzuia mimba wakati ule?”*** *Wekea alama yote yaliyo sahihi)* | Kidonge  Kitanzi  Sindano  Kipandikizi  Kondomu ya kiume  Kondomu ya kike  Kufunga uzazi kwa mwanamke  Kufunga uzazi kwa mwanaume  Njia ya asili ya uzazi wa mpango ya unyonyeshaji  Kumwaga mbegu za kiume nje  Hakuna njia yoyote  Nyingineyo (Taja):  ________________________ | 1  2  3  4  5  6  7  8  9  10  11  12  0  96 |  |
| SW304a | **ANGALIA SW302 NA SW304. ULIZA SWALI HILI KWA WANAWAKE WALE WANAOTUMIA NJIA ZA KISASA ZISIZO ZA KIZUIZI TU.(I.E. KIDONGE, KITANZI, SINDANO,KIPANDIKIZI, AU KUFUNGA UZAZI)**  Ulipatia wapi kidonge/Kitanzi/sindano/kipandikizi/kufunga uzazi? | Eneo la serikali la kutolea huduma za VVU  Eneo la serikali la kutolea huduma za afya ya mama na mtoto/uzazi wa mpango  Kituo binafsi cha kutolea huduma ya VVU  Kituo binafsi cha kutolea huduma ya afya ya mama na mtoto/uzazi wa mpango  Famasi binafsi  Duka lingine  Mfanyakazi wa afya ya jamii  Rafiki/ndugu  Lingine (Taja):  ­­­­­­­­­­­­­­­­­­­­_________________________ | 1  2  3  4  5  6  7  8  96 | 🡪SW305  🡪SW305  🡪SW305  🡪SW305🡪SW305  🡪SW305  🡪SW305  🡪SW305 |
| SW304b | **ANGALIA SW302 NA SW304. ULIZA SWALI HILI KWA WANAWAKE WASIOTUMIA NJIA ZA KISASA ZISIZO ZA KIZUIZI TU KWA MFANO KIDONGE, KITANZI, SINDANO,KIPANDIKIZI, AU KUFUNGA UZAZI)**  Pamoja na kondomu, kuna njia nyingine imara za kuzuia mimba, kama vile kidonge, njia ya sindano, kipandikizi, na kufunga kizazi. Unaweza kuniambia ni kwa nini uliamua kutokutumia mojawapo kati ya njia hizi imara?  *(Usisome hizo njia mbadala, Rekodi tu majibu yanayotajwa na mshiriki.)* | ***Sababu zihusianazo na afya ya uzazi***  Hufanya ngono mara chache  Ukomo wa uwezo wa kuzaa  Hana uwezo wa kupata mimba  Hajatoa damu za kila mwezi tangu mara ya mwisho kujifungua  Kunyonyesha  Mpango wa Mungu  Anataka kupata mimba  ***Kikwazo cha kutumia***  Yuko kinyume na njia hizi  Mwenza/wenza wake yuko/wako kinyume  Watu wengine wako kinyume  Kikwazo cha dini  ***Kukosa ufahamu***  Hajui njia hizi  Hajui mahali pa kupatia huduma hizi  ***Sababu zitokanazo na njia zenyewe***  Madhara/sababu za kiafya  Ukosefu wa namna ya kuzipata/mbali sana  Zina gharama sana  Njia unayotaka haipo  Njia kama hizo hazipatikani  Sio rafiki kwa matumizi  Hukingana na mfumo wa kawaida wa mwili  Nyinginezo (Eleza):  ­­­­_________________________ | 1  2  3  4  5  6  7  8  9  10  11  12  13  14  15  16  17  18  19  20  96 |  |

**SEHEMU 4: NIA YA UZAZI NA UPATAJI MIMBA ULIO SALAMA**

| **Na.** | **Swali** | **Ngazi za alama** | **Alama** | **Rejea** |
| --- | --- | --- | --- | --- |
| SW405 | Kwa sasa unajaribu kupata mimba? | Ndiyo  Hapana | 1  0 | 🡪SW407 |
| SW406 | Unajaribu kupata mtoto na nani? | Mume wa sasa/rafiki wa kiume  Mume wa zamani/rafiki wa kiume wa zamani  Mteja  Mwingine (Eleza):  ________________ | 1  2  3  4  96 |  |
| SW407 | Ungependa kuja kuwa na mtoto (au mwingine) siku moja, au unapendelea kutokuwa na hata mmoja (zaidi)? | Kuwa na mtoto (mwingine)  Nyongeza hapana/Hakuna  Anasema hawezi kushika mimba  Sijui | 1  2  3  98 | 🡪SW409  🡪SW410a  🡪SW409 |
| SW408 | Ni muda gani ungependa kusubiri kuanzia sasa kabla ya kuzaliwa mtoto/mwingine? | Ndani ya mwaka mmoja  Ndani ya miaka miwili  Zaidi ya miaka miwili toka sasa  Sijui | 1  2  3  98 |  |
| SW409 | Ukipata mimba sasa hivi, utajisikiaje? Utajisikia vibaya sana, vibaya kidogo, utafurahi kidogo, au utafurahi sana? | Nitajisikia vibaya sana  Nitajisikia vibaya kidogo  Sitajisikia vibaya wala sitafurahi  Nitafurahi kidogo  Nitafurahi sana | 1  2  3  4  5 |  |
|  | Watu wengi wanaofikiria kuwa na watoto wameathiriwa na VVU. Kwa wanandoa ambao mmoja au wote wawili wanaishi na VVU, ni njia gani ambazo umezisikia kwa wanandoa za kupata mimba bila ya kuambukizana au kumwambukiza mtoto? Katika kila njia zifuatazo, tafadhali nijulishe kama una ufahamu wa kila mkakati:  ***[Usisome majibu – weka alama katika yote yanayohusika]*** | | | |
| SW410a | Dawa za kupunguza makali ya VVU zitumiwazo na mwenza mwenye VVU | Natambua  Sitambui  Sijui | 1  0  98 |  |
| SW410b | Dawa za kuzuia maambukizi ya VVU zitumiwazo na mwenza asiye na VVU (Kinga ya Maambukizi ya VVU au PrEP) | Natambua  Sitambui  Sijui | 1  0  98 |  |
| SW410c | Dawa za kupunguza makali ya VVU zitumiwazo na wanawake katika kipindi cha ujauzito | Natambua  Sitambui  Sijui | 1  0  98 |  |
| SW410d | Kupandikiza. Hii ni pale ambapo mwanaume anamwaga shahawa kwenye kondomu au chombo fulani na baadae kuweka shahawa hizo kwenye uke wa mwanamke. | Natambua  Sitambui  Sijui | 1  0  98 |  |
| SW410e | Wakati muafaka kwa ngono bila kinga. Huu ni wakati ambao wanandoa hufanya ngono zembe katika siku chache za mwezi ambazo mwanamke ana uwezo zaidi wa kushika mimba | Natambua  Sitambui  Sijui | 1  0  98 |  |
| SW410f | Kuosha mbegu za kiume. Hii ni teknolojia ya kusafisha mbegu za mwanaume kuondoa VVU. | Natambua  Sitambui  Sijui | 1  0  98 |  |
| SW410g | Mfadhili wa mbegu za kiume. Hii inahusisha mwanaume asiye naVVU kufadhili mbegu zake ili ziende kumpa mimba mwanamke | Natambua  Sitambui  Sijui | 1  0  98 |  |
| SW411 | Uko na utayari kiasi gani kujifunza kuhusu jinsi ambavyo wanawake wanaoishi na VVU wanavyoweza kujaribu kupata mimba bila kumuambukiza mpenzi au mtoto? | Siko tayari kabisa  Nina utayari kidogo  Niko tayari kabisa | 0  1  2 |  |
| SW412 | Je, mtoa huduma ya afya alishawahi kukupa wewe ushauri au huduma kuhusu jinsi wanawake wanaoishi na VVU wanavyoweza kushika mimba na kuwa na mimba salama? | Ndiyo  Hapana  Sijui namna | 1  0  98 |  |

**SEHEMU 5: TATHMINI YA HUDUMA ZA UZAZI WA MPANGO**

| **No** | **Maswali na vikaguzi** | **Ngazi za Alama** | **Alama** | **Rejea** |
| --- | --- | --- | --- | --- |
| SW501a | Je mtoa alikuuliza kama umepata mtoto ndani ya miezi sita iliyopita? | Hapana  Ndiyo  Sijui | 0  1  98 | 🡪SW501f |
| SW501b | Je mtoa huduma alikuuliza kama unamnyonyesha mtoto wako kikamilifu au kidogo kidogo? | Hapana  Ndiyo  Sijui | 0  1  98 |  |
| SW501c | Je mtoa huduma alikuuliza kama kutokwa na damu yako ya kila mwezi kumerejea? | Hapana  Ndiyo  Sijui | 0  1  98 |  |
| SW501d | Je mtoa huduma alikuuliza kama umejinyima kufanya ngono ya ukeni tangu kujifungua? | Hapana  Ndiyo  Sijui | 0  1  98 |  |
| SW501e | Je mtoa huduma alikuuliza kama umejinyima ngono ya ukeni tangu kurudi kwa damu za kila mwezi? | Hapana  Ndiyo  Sijui | 0  1  98 |  |
| SW501f | Je mtoa huduma alikuuliza kama ulishakuwa na mtoto katika kipindi cha wiki nne zilizopita? | Hapana  Ndiyo  Sijui | 0  1  98 |  |
| SW501g | Je mtoa huduma alikuuliza kama kutokwa na damu yako ya mwisho ya kila mwezi kulianza ndani ya kipindi cha siku saba zilizopita? | Hapana  Ndiyo  Sijui | 0  1  98 |  |
| SW501h | Je mtoa huduma alikuuliza kama uliharibikiwa na mimba au umetoa mimba katika kipindi cha siku saba zilizopita? | Hapana  Ndiyo  Sijui | 0  1  98 |  |
| SW502 | Je mtoa huduma alikuuliza kama kwa sasa unajaribu kupata mimba? | Hapana  Ndiyo  Sijui | 1  0  98 |  |
| SW503 | Je mtoa huduma alikuuliza kama unataka kuja kupata mimba wakati wowote baadae? | Hapana  Ndiyo  Sijui | 1  0  98 |  |
| SW504 | Je mtoa huduma alijadili na wewe jinsi ya kupata mimba wakati ukipunguza hatari ya kusambaza VVU kwa mpenzi wako au mtoto wako? | Hapana  Ndiyo  Sijui | 0  1  98 |  |
| SW505 | Je mtoa huduma alijadili na wewe athari ya VVU juu ya uzazi wako (yaani VVU hupunguza rutuba)? | Hapana  Ndiyo  Sijui | 1  0  98 |  |
| SW506 | Je mtoa huduma kuuliza kama kwa sasa unatumia mbinu yoyote ya kuzuia mimba? | Hapana  Ndiyo  Sijui | 1  0  98 |  |
| SW507 | Baadhi ya wanawake wanaweza kutumia mbinu mbalimbali za kuzuia mimba na wapenzi mbalimbali. Kwa mfano, wanaweza kutumia kondomu ili kuzuia mimba na wateja, lakini wanaweza wasitumie kondomu na marafiki zao wa kiume. Je mtoa huduma alikuuliza kuhusu kama matumizi yako ya njia mbalimbali za uzazi wa mpango ni tofauti kwa wapenzi tofauti? | Hapana  Ndiyo  Sijui | 1  0  98 |  |
| SW508 | Je mtoa huduma alikuuliza kama kwa sasa unatumia mbinu yoyote ya kuzuia mimba kwa wateja wako wasio wa kulipa? | Hapana  Ndiyo  Sijui | 1  0  98 |  |
| SW509 | Je mtoa huduma alikuuliza kama umekuwa ukipata matatizo yoyote na njia (mbalimbali) ya kuzuia mimba unayotumia? | Hapana  Ndiyo  Sijui | 1  0  98 |  |
| SW510 | Je mtoa huduma alikuuliza kama ulikuwa na upendeleo kwa mbinu yoyote ya kuzuia mimba? | Hapana  Ndiyo  Sijui | 1  0  98 |  |
| SW511 | Ni ipi kati ya mbinu zifuatazo ambazo mtoa huduma alijadili na wewe?  ***SOMA MAJIBU KWA SAUTI NA CHAGUA YOTE YANAYOSTAHILI.*** | Kidonge  Kitanzi  Sindano  Kipandikizi  Kondomu ya kiume  Kondomu ya kike  Kufunga uzazi kwa mwanamke  Kufunga uzazi kwa Mwanaume  Njia ya asili ya uzazi wa mpango ya unyonyeshaji  Kumwaga mbegu za kiume nje  Uzazi wa mpango wa dharura  Hakuna mbinu iliyojadiliwa  Nyingine (taja):  ________________________ | 1  2  3  4  5  6  7  8  9  10  11  12  0  96 |  |
| SW512 | Je mtoa huduma alitaja madhara ya mbinu mbalimbali na wewe? | Ndiyo  Hapana  Sijui | 1  0  98 |  |
| SW513 | Je mtoa huduma alijadili jinsi ambavyo madhara haya yanavyoweza kuathiri uwezo wako wa kuuza ngono? | Ndiyo  Hapana  Sijui | 1  0  98 |  |
| SW514 | Je mtoa huduma alijadili jinsi dawa za kurefusha maisha zinavyoweza kuathiri jinsi mbinu za kuzuia mimba zinavyofanya kazi? | Ndiyo  Hapana  Sijui | 1  0  98 |  |
| SW515 | Je mtoa huduma alizungumza na wewe kuhusu kumwambia (m)wapenzi wako kuhusu hali yako ya VVU (yaani, kutoa taarifa)? | Ndiyo  Hapana  Sijui | 1  0  98 |  |

**SEHEMU 6. TABIA ZIHUSIANAZO NA VVU**

| SW601 | Unakumbuka kiwango chako cha CD4? | 100 au pungufu  101-200  201-300  301-500  Zaidi ya 500  Sikumbuki | 1  2  3  4  5  98 |  |
| --- | --- | --- | --- | --- |
| SW602 | Je ulishapimwa uwingi wa virusi (viral load) katika kipindi cha miezi 6 iliyopita? | Ndiyo  Hapana | 1  0 |  |
| SW603 | Je, kwa sasa unatumia matibabu ya kuounguza makali ya VVU? | Hapana  Ndiyo  Sijui | 0  1  98 | 🡪SW605 |
| SW604 | Katika kipindi cha siku 7 zilizopita, ni siku ngapi umekosa kutumia vidonge vyako vyote? | Hakuna siku  Siku moja  Siku mbili  Siku tatu | 0  1  2  3 |  |
| SW605 | Ni kwa mara ngapi unamwonaga daktari wako wa VVU? | Kila mwezi 1  Kila baada ya miezi 3  Kila baada ya miezi 4-6  Nyingine_________ | 1  2  3  96 |  |
| SW606 | Mbali na watoa huduma wako wa VVU (madaktari / wauguzi), je, ulishamwambia mtu yeyote kwamba unaishi na VVU? | Ndiyo  Hapana | 1  0 | 🡪Mwisho |
| SW607 | Kama ndiyo, ulimweleza nani? | Mteja anayelipia ngono  Wapenzi/wenza wasiolipia ngono  Wazazi  Ndugu  Wanachama wa kikundi cha ushirika  Waelimishaji rika  Nyingine (Taja) __________ | 1  2  3  4  5  6  96 |  |

***Asante kwa muda wako.***
